# Editorial: Magnetic Resonance Spectroscopy of GABA and Glutamate in Mental Health

**DOI:** 10.3389/fpsyt.2022.866356

**Published:** 2022-03-11

**Authors:** Maria Concepcion Garcia Otaduy

**Affiliations:** LIM44, Instituto e Departamento de Radiologia, Faculdade de Medicina, Universidade de São Paulo, São Paulo, Brazil

**Keywords:** magnetic resonance spectroscopy, GABA, glutamate, mental health, MEGA-PRESS

This Research Topic presents a variety of studies that illustrate the vast potential that magnetic resonance spectroscopy (MRS) offers to investigate glutamate (Glu) and/or gamma aminobutyric acid (GABA) metabolism related to different psychiatric conditions. GABA is the primary inhibitory neurotransmitter in the brain, and Glu the principal excitatory transmitter. For several aspects of mental health, it is important that there is a balance between both of these molecules, and it is already known that in several psychiatric conditions (i.e., schizophrenia, depression, bipolar disorder, autism), one or both of these metabolites are altered. Currently, MRS is the only non-invasive neuroimaging technique that allows *in vivo* investigation of glutamatergic and GABAergic abnormalities within brain regions of interest.

Four thorough reviews, eight original research papers, three brief research reports, and one methods paper compose the present Research Topic, in an attempt to bring a diverse overview about the current possibilities to study the roles of GABA and Glu in mental health by MRS.

The review *Glutamate and GABA Homeostasis and Neurometabolism in Major Depressive Disorder* presents a comprehensive introduction about the role of Glu and GABA in brain homeostasis and metabolism, and describes briefly how these metabolites can be measured by 1H- and 13C-MRS. It then summarizes how these processes appear to be unbalanced in several psychiatric diseases, with an emphasis in major depressive disorder, and how antidepressant treatment affects the glutamatergic and GABAergic systems (Sarawagi et al.).

Four of the original research articles in this topic focus on investigating Glu/Glx and/or GABA alterations in different psychiatric disorders: Obsessive-compulsive disorder, schizophrenia, bipolar disorder, and depression. Interestingly all of them studied a large group of patients (≥40), allowing the researchers to investigate possible associations with disease duration or other clinical variables, in an attempt to contribute robust and reproducible results to the field.

The methodologies used in these four studies are very diverse. In *Higher levels of pro-inflammatory cytokines are associated with higher levels of glutamate in the anterior cingulate cortex in depressed adolescents*, the authors used a single voxel PRESS sequence, a robust and fast acquisition, with the main focus of quantifying Glu and ascorbate (Ho et al.). In *Increased Glutamate plus Glutamine in the Right Middle Cingulate in Early Schizophrenia but not in Bipolar Psychosis: A Whole Brain 1H-MRS Study*, a more time-demanding (about 17 min) 3D-MRSI EPSI sequence was used, which allows the construction of whole-brain metabolite maps that can be compared between groups to identify the location of individual voxels presenting statistical significant differences (Bustillo et al.). This is a very interesting approach, as metabolic changes can occur at different regions, and conventional single voxel spectroscopy also suffers from partial volume effects due to larger voxel sizes. But none of these techniques at 3T allow for quantification of GABA. For this purpose the use of editing techniques like MEGA-PRESS or 2D-JPRESS, as adopted in *Lower ventromedial prefrontal cortex glutamate levels in patients with obsessive-compulsive disorder*, is necessary (Batistuzzo et al.). At ultra-high fields (≥7T), the chemical shift dispersion is large enough to enable the distinction of GABA, Glu, and glutamine from the other overlapping metabolites, without the need of using editing pulses, as shown in *Metabolite alterations in adults with schizophrenia, first degree relatives, and healthy controls: a multi-region 7T MRS study* (Wijtenburg, Wang, et al.). There is no doubt that the MRS field in psychiatry will hugely benefit from the slowly increasing number of MRS studies conducted at 7T.

In *GABA, glutamate, and neural activity: a systematic review with meta-analysis of multimodal 1H-MRS-fMRI studies*, the authors successfully gather evidence to elucidate the relationship between GABA/Glu and brain activation, in the resting state and during stimulation (Kiemes et al.).

This Research Topic also includes several examples of how multimodal 1H-MRS-fMRI studies can enhance our understanding of brain function and metabolism. In *Simultaneous measurement of the BOLD effect and metabolic changes in response to visual stimulation using the MEGA-PRESS sequence at 3 T*, the authors implement an interesting method, using an MEGA-edited GABA-PRESS sequence at 3T, to measure simultaneously the BOLD effect from the linewidth of the unsuppressed water spectrum, and the changes in both Glx and GABA levels in the occipital cortex during visual stimulation (Dwyer et al.). The method proved to be very reliable for measuring the BOLD effect from the water signal linewidth, but still needs further validation for proper metabolite quantification. In *Multimodal Neuroimaging Study of Visual Plasticity in Schizophrenia*, the authors are able to demonstrate, by using a paradigm-based fMRI experiment, that visual plasticity in schizophrenia is impaired and that it is related to basal GABA levels (Wijtenburg, West, et al.). In a different approach, the authors of *Glutamate- and GABA-modulated connectivity in auditory hallucinations—a combined resting state fMRI and MR spectroscopy study* use functional connectivity results from resting state fMRI data to show that its association with metabolic levels of Glu and GABA is different according to auditory verbal hallucination severity in patients with psychosis (Weber et al.). And in *Unaltered Brain GABA concentrations and Resting fMRI Activity in Functional Dyspepsia with and without Comorbid Depression*, the authors also use resting state fMRI data to evaluate possible differences in brain activation and metabolism between patients with functional dyspepsia with and without major depression disorder (Mak et al.).

The review *Local and Interregional Neurochemical Associations Measured by Magnetic Resonance Spectroscopy for Studying Brain Functions and Psychiatric Disorders* presents the basis for neurochemical association of glutamate with GABA, NAA, and glutamine and discusses the importance of observing this association, as it can be altered in specific mental health disorders (Shen et al.). In such an endeavor, they highlight the role of spectral editing spectroscopy techniques in eliminating unwanted correlations caused by spectral overlapping. The paper ends with a review of the methods available to correctly quantify the strength of these neurometabolic associations, not only locally but also between different brain regions by using chemical shift imaging (CSI). For CSI, the combination of ultra-high field strengths (7T) is desired, as it allows the researchers to resolve Glu and GABA without the use of highly selective editing pulses and simplifies the multivoxel acquisition process.

In the review paper *Elevated brain glutamate levels in bipolar disorder and pyruvate carboxylase-mediated anaplerosis*, the authors show supporting evidence for the interesting hypothesis that elevated Glu levels in bipolar disorder can be explained by increased pyruvate carboxylase-mediated anaplerosis in the brain (Shen and Tomar).

This Research Topic includes also three brief research reports that bring very promising preliminary results about the use of MEGA-PRESS at 3T in longitudinal studies to monitor treatment or intervention effects: *Intermittent theta-burst stimulation transcranial magnetic stimulation increases GABA in the medial prefrontal cortex: a preliminary sham-controlled magnetic resonance spectroscopy study in acute bipolar depression* (Diederichs et al.); *Left dorsolateral prefrontal cortex Glx/tCr predicts efficacy of high frequency 4- to 6-week rTMS treatment and is associated with symptom improvement in adults with major depressive disorder: Findings from a pilot study* (Bhattacharyya et al.); and *Cortical Glutamate and GABA changes during Early Abstinence in Alcohol Dependence and their Associations with Benzodiazepine Medication* (Wang et al.).

As a final remark, the importance of the methods paper *Feasibility of measuring GABA levels in the upper brainstem in healthy volunteers using edited MRS* needs to be highlighted, since it opens up a new scope of research to address some important brain functions in the brainstem, and their relation to mental health (Song et al.). The upper brainstem contains many integrative nuclei that mediate physiological functions, known to be disrupted in neurodegenerative diseases. In this paper, the authors show the feasibility of acquiring high-quality 3T GABA-specific MEGA-PRESS spectra in the upper brainstem.

Throughout the Research Topic, methodological rigor, especially in relation to spectroscopy acquisition techniques, was one of the top priorities in the preparation of this volume, as we understand this is necessary for obtaining meaningful results that can contribute to the field. In the last few years, the MRS scientific community has been making a huge effort to establish useful guidelines for MRS acquisition and reports (1–5). Hopefully this will also facilitate large-scale collaborative MRS studies about Glu and GABA in psychiatric conditions similar to that proposed by the ENIGMA consortium for genetic and neuroimaging data.

## Author Contributions

The author confirms being the sole contributor of this work and has approved it for publication.

## Conflict of Interest

The author declares that the research was conducted in the absence of any commercial or financial relationships that could be construed as a potential conflict of interest.

## Publisher's Note

All claims expressed in this article are solely those of the authors and do not necessarily represent those of their affiliated organizations, or those of the publisher, the editors and the reviewers. Any product that may be evaluated in this article, or claim that may be made by its manufacturer, is not guaranteed or endorsed by the publisher.
